# In-Stent Restenosis and a Drug-Coated Balloon: Insights from a Clinical Therapeutic Strategy on Coronary Artery Diseases

**DOI:** 10.1155/2020/8104939

**Published:** 2020-10-25

**Authors:** Dai-Min Zhang, Shaoliang Chen

**Affiliations:** Department of Cardiology, Nanjing First Hospital, Nanjing Medical University, Nanjing, Jiangsu 210006, China

## Abstract

Coronary heart disease is a major cause of death and disability in developed countries. Stent implantation has become an efficacious treatment for a culprit lesion vessel of the coronary artery. However, 10%–20% restenosis is still an important complication that restricts the clinical safety and efficacy of drug-eluting stents. In-stent restenosis may lead to the recurrence of major cardiovascular adverse events, including angina pectoris, acute myocardial infarction, and even sudden cardiac death. These events are currently serious problems that occur after coronary stent implantation. Clinical physicians face a difficult choice for in-stent restenosis treatment. Recent studies indicate that a drug-coated balloon has promising clinical efficacy similar to the drug-eluting stents for treating coronary in-stent restenosis. Therefore, in this study, we highlight the progress of coronary intervention and the use of drug-coated balloons in the treatment of in-stent restenosis (ISR).

## 1. Introduction

Coronary heart disease (CHD) has become the “first killer” that endangers human health. CHD weakens the heart muscle and leads to heart failure and arrhythmias. Percutaneous coronary intervention (PCI) helps in recovering the coronary flow and has become an efficacious treatment for revascularization of the blocked coronary artery.

PCI includes percutaneous transluminal coronary angioplasty (PTCA), stent implantation, and drug-coated balloons (DCBs). The rapid development of stent bioengineering technology and drug carriers has improved the safety and efficacy of stent treatment [1, 2]. Compared with PTCA in which restenosis occurs at the rate of up to 50% [3], the rate of in-stent restenosis (ISR) in patients with drug-eluting stents (DESs) decreased significantly; however, 10%–20% restenosis is still a major complication, restricting the clinical safety and efficacy of DESs [4, 5]. ISR may lead to a recurrence of major adverse cardiovascular events (MACEs), including angina pectoris, acute myocardial infarction, and even sudden cardiac death.

No stents were available in the early 1970s. Although balloon dilation could reduce coronary artery stenosis, *owing to the property of elastic recoil and intimal tear of the vessel wall, vascular restenosis may reoccur again*. The vascular retraction rate after simple balloon dilatation angioplasty was as high as 30% to 60% 3 to 6 months postoperatively. In the 1980s, the emergence of bare-metal stents (BMSs) led to a 30% incidence of restenosis [6, 7]. Recent DESs combined with antiproliferative drugs, such as paclitaxel and sirolimus, reduced the incidence of ISR to less than 10% [8]. Although the incidence of ISR is gradually declining, still it cannot be completely prevented.

Although ISR is much less common with the use of DESs than BMSs, the number of stents implanted in interventional practice means that the treatment of ISR remains an important clinical challenge [9]. Clinical physicians face a difficult choice for ISR treatment. Accumulative evidence indicates that drug-coated balloon (DCB) angioplasty, as a similar new DES treatment for coronary ISR, have been considered as an alternative option for treating coronary ISR [10–12], avoiding repeated stenting after ISR that leads to expose the patient to cost and risk of a prolonged DAPT. Therefore, in this study, we highlight the progress of coronary intervention and the use of DCBs in the treatment of ISR.

## 2. In-Stent Restenosis

ISR is defined as new proliferative lesions of more than or equal to 50% of the lumen diameter, involving either the stent segment or adjacent 5 mm segments on both sides of the stent by coronary angiography (CAG) [9, 10]. After a successful PCI procedure, coronary stents can fail to maintain vessel patency as a result of either restenosis or thrombosis; restenosis is a gradual re-narrowing of the stent segment that mostly occurs between 3 and 12 months after stent implantation. Restenosis usually presents as recurrent angina but can present as an acute myocardial infarction in approximately 10% of patients [13]. Restenosis can usually be managed by repeat percutaneous revascularization.

The patterns of ISR lesions can be divided into two broad categories [10]: focal—lesions localized within the stent that are <10 mm in length are said to be Type I lesions and can be focal single or focal multiple—and diffuse—diffuse lesions are >10 mm in length and may or may not be confined to the edges of the stent. Type II diffuse lesions are confined to the stent edges, Type III diffuse lesions overhang the stent edges, and Type IV diffuse lesions completely occlude the stent.

### 2.1. Pathology of ISR

Restenosis was understood to be the result of exuberant smooth muscle cell proliferation and macrophage infiltration inside the stent. Arterial vessels are abundant in elastic fibers that provide vessels with recoiling properties after they are distended by any means [14]. Recoiling occurs within seconds to minutes after the procedure. ISR is mainly caused by neointimal hyperplasia, which occurs in response to local arterial injury sustained during PCI, leading to complex inflammatory and reparative processes. Neointimal hyperplasia first develops as damage to the arterial wall, followed by platelet aggregation at the site of the injury, recruitment of inflammatory cells, proliferation and migration of vascular smooth muscle cells, and collagen deposition [15].

Deployment of a stent at the lesion site requires inflation of a balloon apposing it in approximation with the vessel wall, thereby stretching the plaque and vessel wall layers [15, 16]. Intima and media tears give rise to a complex inflammatory response; this phenomenon, via multiple mechanisms, can lead to excessive neointimal proliferation, thereby decreasing the minimal luminal diameter (MLD) [16]. Neointimal proliferation is a process by which smooth muscle cells and myofibroblasts are mobilized from the media and adventitia of vessel walls, respectively. Redistribution and overgrowth of smooth muscle cells on stent struts cause luminal loss. This process takes months and is almost complete at 6 months; after 6 months, there is a shift in the cellular distribution that causes the collagen and proteoglycan matrix to become the major component of the growing lesion. Interestingly, this shift in cellularity leads to a relative decrease in late luminal loss as a result of the shrinkage in the lesion width. This process occurs from 6 months to 3 years.

### 2.2. Neoatherosclerosis

Neoatherosclerosis is defined as the accumulation of lipid-laden macrophage foam cells within the neointima of stented arteries, with or without necrotic core formation or calcification [17]. Mechanical injury to arterial vessels as a result of the arterial wall stretching with a https://en.wikipedia.org/wiki/Ballooncatheter results in the recruitment of cells, such as monocytes, https://en.wikipedia.org/wiki/Macrophage, and https://en.wikipedia.org/wiki/Neutrophil, to the site of the injury [16]. While atherosclerosis in native arteries develops over decades, neoatherosclerosis seems a much faster process. This might be due to loss of or damage to endothelial function induced by PCI; moreover, antiproliferative drugs in DESs might even worsen the restoration of a competent endothelium [18]. Intravascular imaging, OCT (optical coherence tomography) in particular, can provide clues about the underlying pathology in stent failure; however, results might not always be easy to interpret.

### 2.3. Clinical Management

The introduction of DESs proved to be an important step forward in reducing the rate of restenosis and target lesion revascularization (TLR) after PCI. However, the rapid implementation of DESs in standard practice and expansion of the indications for percutaneous coronary intervention in high-risk patients and complex lesions also introduced a new problem, specifically, DES-ISR. DES-ISR occurs in 3% to 20% of patients, depending on the patient and lesion characteristics and DES type. The clinical presentation of DES-ISR can be recurrent angina, but some patients present with acute coronary syndrome (ACS) [1, 6]. The mechanisms of DES-ISR can be biological, mechanical, or technical; its pattern is predominantly focal. Intravascular imaging can assist in defining the mechanism and selecting treatment modalities. Based on the current available evidence, intravascular imaging will help in selecting optimal approaches to treat DES restenosis [9].

Among the predictors of patient-related factors, a history of ISR is the major factor in determining the chances of developing stent restenosis or TLR [5]. To date, considerable evidence is available that supports a refined stent structure with improved results and outcomes in terms of ISR. Second-generation DESs are improved versions and have decreased late luminal loss and ISR compared with first-generation stents. Compared with first-gen DESs, the second-gen devices have different drugs and polymers, along with improved platform design and reduced strut thickness.

In addition, high-risk factors for cardiovascular diseases include hypertension, diabetes mellitus, and hyperlipidemia. Neointimal hyperplasia is considered to be one of the pathogenic mechanisms of ISR. DESs achieve a good therapeutic effect because the antiproliferative drugs on its surface inhibit neointimal hyperplasia. However, prolonged and slow drug release of DESs can lead to delayed vascular endothelialization [5]. The challenge now is to develop the correct combination of drugs and coating to eliminate and not just reduce ISR [19]. DCBs offer the possibility to achieve fast-released and rapidly absorbed antiproliferative drugs without adding more metallic struts.

## 3. Evidence-Based Practice of Treating ISR Using DCBs

The treatment options for ISR include PCI with DESs, DCBs, or PTCA. DCBs and second-generation DESs were equally effective in treating coronary ISR, according to a new systematic review and meta-analysis [20]. Paclitaxel and rapamycin analogues have been proven successful in preventing neointimal hyperplasia and ISR. Different characteristics of these agents may affect their clinical efficacy. Paclitaxel is a diterpenoid compound isolated from the Pacific yew tree. Due to its high lipophilic characteristics, paclitaxel promotes rapid cellular uptake. Paclitaxel inhibits mitotic progression and cell proliferation in the G0-G1 and G2-M phases of the cell cycle [21]. Sirolimus, a rapamycin analogue, forms a complex with the cytosolic immunophilin, FK506 binding protein-12, which in turn blocks the activation of the cell-cycle-specific kinase, mammalian target of rapamycin (mTOR). mTORC1 regulates translation, transcription, cell cycle progression, and survival through the phosphorylation of p70 56 kinase and 4E-BP1 [22–25].

Drug-coated balloon angioplasty is similar to plain old balloon angioplasty, with the addition of an antiproliferative medication coating on the balloon, which helps reduce and prevent restenosis [26–28]. The drug coating comprises an active drug and a carrier. The active drugs are paclitaxel and rapamycin. The current DCB prevents the premature release of drugs before the balloon catheter is positioned at a target site and promotes the drugs that are quickly released from the surface of the balloon and absorbed by the target tissue.

DCBs were used to treat coronary ISR [29]. Recent clinical trials on DCBs are summarized in Table 1. Scheller et al. [30] reported that a paclitaxel-eluting balloon (PEB) significantly reduced the incidence of ISR compared with POBA in porcine coronary arteries. In a study by Scheller et al. [31], the LLL of PEBs was significantly lower than that of POBA by CAG after 6 months of follow-up; the incidences of ISR and MACEs were also significantly lower than that of POBA. Furthermore, long-term follow-up results also showed a good therapeutic effect with PEBs [32].

A total of 131 patients with BMS-ISR were enrolled in a randomized, nonblinded trial conducted at 10 German cardiovascular centers (PEPCAD II experiment) [33], including 66 patients with PEBs and 65 patients with a paclitaxel-eluting stent (PES). After six months of follow-up, CAG showed that the late lumen loss (LLL) in the PEB group was lower than that in the PES group, with a borderline difference in restenosis. An as-treated analysis revealed that all DCBs crossed the lesion, whereas four DESs failed to cross the lesion. At the 12-month follow-up, an intention-to-treat analysis revealed that the lesion-related rate of MACEs was 7.6% in the DCB group vs. 16.9% in the DES group; at 36 months, these rates were 9.1% in the DCB group versus 18.5% in the DES group. These disparities were largely attributed to the decreased target lesion revascularization (TLR) in the DCB group compared with DES group.

The PEPCAD-DES study [35] compared the DES-ISR results between DCBs and POBA. The six-month follow-up showed that the LLL in the DES group was superior to that in the POBA group. The ISAR-DESIRE3 trial [34] compared the therapeutic outcomes of DES-ISR with DCBs, DESs, and POBA. The six-month follow-up showed that the DCB and DES groups had similar treatment outcomes and were superior to the POBA group. In patients with coronary bare-metal ISR, the use of a paclitaxel-coated balloon catheter appears to yield superior long-term angiographic and clinical outcomes compared with a paclitaxel-eluting stent according to 3-year follow-up data from the PEPCAD II ISR study.

Prospective, multicenter, and randomized controlled studies from China [36] showed that 220 patients with DES-ISR were enrolled in the PEPCAD China ISR. They randomized patients to receive PCB or PES treatment. After 9 months of follow-up, the LLL of the PCB group was compared with that of the PES group; the 12-month TLRs were 14.5% and 13.6%, respectively. The difference was not statistically significant. Therefore, DCBs and DESs are effective for the treatment of DES-ISR, but DCBs carry out a novel concept for coronary intervention without implantation.

A prospective multicenter trial [37] evaluated the safety and efficacy of Pantera Lux paclitaxel-coated balloons. 1,064 patients were treated for predominantly diffuse and proliferative in-stent restenosis of BMS (BMS-ISR) and DES (DES-ISR), or for de novo lesions. MACEs were 8.5% in the overall, 6.0% in the BMS-ISR, 11.5% in the DES-ISR, and 7.0% in the de novo population at six months, and 15.1%, 11.6%, 20.6%, and 9.4% at 12 months, respectively. Safety and efficacy of the Pantera Lux paclitaxel-coated balloon was confirmed with low major adverse cardiac event rates in patients with in-stent restenosis or de novo lesions.

A prospective single-blind randomized trial conducted in patients with SES restenosis was to investigate the efficacy of a PEB for the treatment of sirolimus-eluting stent (SES) restenosis. At 6-month angiographic follow-up, in-segment late lumen loss was lower in the PEB group than in the BA group. The incidence of recurrent restenosis and target lesion revascularization was also lower in the PEB group than in the BA group. The cumulative MACE-free survival was significantly better in the PEB group than in the BA group. In patients with SES restenosis, PEBs provided much better clinical, angiographic outcomes than conventional BA [38].

A multicenter, prospective study included 2,095 patients with 2,234 lesions at 75 centers and assesses the safety and efficacy of paclitaxel-coated balloon (PCB) angioplasty. The TLR rate was significantly lower in patients with PCB angioplasty for BMS restenosis compared with DES restenosis. These results suggested that PCB angioplasty was more effective in BMS restenosis compared with DES restenosis [39].

Stella et al. [40] assess the safety and efficacy of the second-generation DIOR paclitaxel drug-eluting balloon (DEB) for in-stent restenosis. Following 8 months, in-stent restenosis treatment with the second-gen DIOR DEB is safe and feasible, with high angiographic success and low target lesion revascularization and overall MACE rates.

Hehrlein et al. [41] investigated safety and efficacy of a novel DCB incorporating paclitaxel into a microcrystalline structure by applying the inert excipient butyryl tri-n-hexyl citrate (BTHC). At 6 months, overall LLL showed differences in BMS-ISR and DES-ISR treatment. Application of a novel paclitaxel-coated balloon using BTHC as an excipient in patients with ISR is safe and results in very low LLL and revascularization and MACE rates.

The RIBS IV trial is a prospective multicenter randomized clinical trial comparing DEBs and EES in patients with DES-ISR. The 3-year clinical outcomes indicated that the in-segment minimal lumen diameter was larger in the EES arm. The need for late target lesion revascularization and target vessel revascularization was similar in the 2 arms. Rates of cardiac death, myocardial infarction, and stent thrombosis were also similar in both arms [42].

Based on a large amount of clinical evidence, the European Society of Cardiology and European Society of Thoracic and Cardiovascular Surgery jointly issued the Guideline for Revascularization to classify the evidence of DCBs for treating various types of ISR (including BMS-ISR and DES-ISR) into Class I Type A [43]. China's Food and Drug Administration also approved DCBs as a clinical indication for the treatment of ISR.

Recently, a MagicTouch Sirolimus DCB catheter using Nanolute-based drug delivery platform technology was used for the treatment of coronary ISR. Not only can it facilitate drug transfer to the arterial wall with circumferential coating, but it also improves the drug adhesion and in-tissue bioavailability of sirolimus. Ongoing multicenter, randomized clinical trials were conducted to evaluate the safety and efficacy of ISR treatment with DCBs [44].

## 4. Further Perspective

ISR is the in-stent narrowing of a coronary artery lesion. The mean time from PCI to ISR is 12 months with DESs and 6 months with bare-metal stents (BMSs) [9, 45]. ISR typically presents as recurrent angina. The use of DESs significantly reduced the rate of ISR compared with BMSs. Intravascular ultrasound, optical coherence tomography, and fractional flow reserve are important tools for anatomic and hemodynamic assessments of ISR. The mechanism of stent failure is represented by metallic struts themselves (malapposition and subexpansion), and in these situations, implantation of another stent can be deleterious. Moreover, the underlying pathological mechanism of ISR should probably be taken into account. We do not know exactly if DCBs are equally effective in treating ISR due to neointimal hyperplasia compared with neoatherosclerosis, although we can hypothesize that they might be more effective in the former case.

So far, it is still unclear whether the correction of artery stenosis by stents is fully hemocompatible or their implantation causes alterations at the level of the plasma membrane in red blood cells. Basoli et al. [46] reported that full hemocompatibility of stents has not yet been reached after stent implantation. There are some measurable alterations in the passive electrical behavior of the red blood cell membrane induced by the presence of the stent. The passive electrical properties of the erythrocyte membrane before and after stent insertion measured by dielectric relaxation spectroscopy play import roles in investigating the hemocompatibility of a medical device at a cell membrane level. Those factors may trigger the change of microenvironment around stents, eventually contribute to ISR.

Although recent alarming data from a large meta-analysis in patients with peripheral arterial diseases (PADs) indicated that the use of paclitaxel-coated balloons or paclitaxel DESs was associated with a higher risk of late mortality compared with alternative therapeutic modalities, these concerns did not influence the use of drug-coated balloons in coronary arteries [47, 48]. Multiple randomized clinical trials and meta-analyses have shown the safety and efficacy of PCBs in patients with ISR [49, 50].

Compared with reimplantation of a DES, a DCB's advantages include three aspects [12, 27, 29, 51]. First, it can evenly distribute antiproliferative drugs in the vessel wall. It does not cause an endothelial delay as a result of stent dilatation or the uneven distribution of metals, and it does not leave a residue of the metal grid of the stent, avoiding the endothelium inflammatory response, reducing the incidence of advanced thrombosis, and reducing the DAPT time. Second, DCB can avoid repeated placement of stents, reducing the impact on the anatomy of the coronary artery and retaining the opportunity for patients to be treated again. Finally, the DCB is also suitable for patients with abnormal coagulation and for patients with a high risk of bleeding.

The emergence of DCBs can be described as landmark development of cardiac interventional technology and optimize clinical outcomes in these specific lesions. Furthermore, the DCB may become a viable alternative treatment option for the inhibition of coronary ISR and subsequent revascularization, as it allows the local release of a high-concentration antirestenosis drug into the coronary vessel without using a metal scaffold or durable polymers. The clinical trial of the drug-coated balloon in the treatment of in-stent restenosis. Inspired by these results, an increasing number of studies have been conducted on different coronary lesion subsets to explore the efficacy of DCBs in a broader range of lesions.

## Figures and Tables

**Table 1:** The clinical trial of the drug-coated balloon in the treatment of in-stent restenosis.

	Trial name	Balloon and groups	Principal findings	Ref.
Scheller et al., 2006	PACCOCATH ISR I	PACCOCATH® DEB (26 cases) vs. Uncoated balloon (26 cases)	At 6-month follow-up, LLL: (0.03 ± 0.48) mm vs. (0.74 ± 0.86) mm (*P*=0.002); TLR 23% vs. 0%	[30]

Scheller et al., 2008; Scheller et al., 2012	PACCOCATH ISR II	PACCOCATH® DEB (54 cases) vs. uncoated balloon (54 cases)	6 or 12 months, LLL: (0.81 ± 0.79) mm vs. (0.11 ± 0.46) mm (*P* < 0.001); 37% vs. 4%	[31, 32]

Unverdorben et al, 2009	PEPCAD II	SeQuent Please (66 cases)/TAXUS stents (65 cases)	6 months, LLL: (0.17 ± 0.42) mm vs. (0.38 ± 0.61) mm (*P*=0.03); 6.3% vs. 15.4%	[33]

Byrne et al. 2013	ISAR-DESIRE3	SeQuent Please (137 cases)/TAXUS stent (131 cases)/common balloon (134 cases)	9 months, in-stent restenosis diameter: 38%:37.4% vs. 54.1% (noninferiority *P*=0.007)	[34]

Rittger et al., 2012	PEPCAD-DES	SeQuent Please (72 cases)/common balloon (38 cases)	6 months, LLL: (0.43 ± 0.61) mm vs. (1.03 ± 0. 77) mm (*P* < 0.001)	[35]

Xu et al., 2014	PEPCAD China ISR	SeQuent Please (110cases)/TAXUS (110 cases)	9 months, LLL: (0.46 ± 0.51) mm vs. (0.55 ± 0.61) mm (*P* noninferiority = 0.0005)	[36]

Toelg et al., 2014	DELUX registry	Pantera Lux DCB (1064 cases)	6, 12 months, MACE: 8.5%, 15.1%	[37]

Habara et al., 2011	Habara et al.	SeQuent Please (25 cases)/common balloon (25 cases)	6 months, LLL: (0.18 ± 0.45) mm vs. (0.72 ± 0.55) mm (*P*=0.001)	[38]

Wöhrle et al., 2012	SeQuent Please World Wide Registry	SeQuent Please (DES-ISR (464 cases)/BMS-ISR (763 cases))	9 months, TLR: 9.6% vs. 3.8% (*P* < 0.001)	[39]

Stella et al., 2011	Valentines I	DIOR II DCB (paclitaxel DES-ISR (34 cases)/everolimus DES-ISR (42 cases))	8 months, MACE: 0 : 23.8% (*P*=0.002)	[40]

Hehrlein et al., 2012	PEPPER	Pantera Lux DES (BMS-ISR (43 cases)/DES-ISR (38 cases))	6 months, LLL: (0.05 ± 0.28) mm vs. (0.19 ± 0.29) mm (*P*=0.001)	[41]

Alfonso et al., 2018	RIBS IV	DCB (154 cases)/EES (155 cases)	3-yr follow-up, MLD: (2.03 ± 0.7) mm vs. (1.8 ± 0.6) mm	[42]

*Note.* DCB, drug-coated balloon; DES, drug-eluting stent; MACE, major adverse cardiac event; LLL, late lumen loss; MLD, minimal lumen diameter; TLR, target lesion revascularization.

## References

[1] Garg S., Bourantas C., Serruys P. W. (2013). New concepts in the design of drug-eluting coronary stents. *Nature Reviews Cardiology*.

[2] Scacciatella P., D’Amico M., Pennone M. (2013). Effects of EPC capture stent and CD34+ mobilization in acute myocardial infarction. *Minerva Cardioangiol*.

[3] Nobuyoshi M., Kimura T., Nosaka H. (1988). Restenosis after successful percutaneous transluminal coronary angioplasty: serial angiographic follow-up of 229 patients. *Journal of the American College of Cardiology*.

[4] Mohan S., Dhall A. (2010). A comparative study of restenosis rates in bare metal and drug-eluting stents. *International Journal of Angiology*.

[5] Park S. J., Kang S. J., Virmani R., Nakano M., Ueda Y. (2012). In-stent neoatherosclerosis. *Journal of the American College of Cardiology*.

[6] Lee M. S., Pessegueiro A., Zimmer R., Jurewitz D., Tobis J. (2008). Clinical presentation of patients with in-stent restenosis in the drug-eluting stent era. *The Journal of Invasive Cardiology*.

[7] Schnorr B., Speck U., Scheller B. (2011). Review of clinical data with Paccocath- coated balloon catheters. *Minerva Cardioangiologica*.

[8] Taniwaki M., Stefanini G. G., Silber S. (2014). 4-Year clinical outcomes and predictors of repeat revascularization in patients treated with new-generation drug-eluting stents. *Journal of the American College of Cardiology*.

[9] Dangas G. D., Claessen B. E., Caixeta A., Sanidas E. A., Mintz G. S., Mehran R. (2010). In-stent restenosis in the drug-eluting stent era. *Journal of the American College of Cardiology*.

[10] Mehran R., Dangas G., Abizaid A. S. (1999). Angiographic patterns of in-stent restenosis. *Circulation*.

[11] Zhu J., Ge J. (2015). GW26-e1581 a meta-analysis of randomized controlled trials of plain old balloon angioplasty versus drug-eluting balloon in patients with in-stent restenosis. *Journal of the American College of Cardiology*.

[12] Alfonso F., Pérez-Vizcayno M. J., García del Blanco B. (2016). Comparison of the efficacy of everolimus-eluting stents versus drug-eluting balloons in patients with in-stent restenosis (from the RIBS IV and V randomized clinical trials). *The American Journal of Cardiology*.

[13] Lee M. S., Banka G. (2016). In-stent restenosis. *Interventional Cardiology Clinics*.

[14] Serrano M. C., Vavra A. K., Jen M. (2011). Poly (diol-co-citrate)s as novel elastomeric perivascular wraps for the reduction of neointimal hyperplasia. *Macromolecular Bioscience*.

[15] Danenberg H. D., Welt F. G. P., Walker M., Seifert P., Toegel G. S., Edelman E. R. (2002). Systemic inflammation induced by lipopolysaccharide increases neointimal formation after balloon and stent injury in rabbits. *Circulation*.

[16] Shah P. K. (2003). Inflammation, neointimal hyperplasia, and restenosis. *Circulation*.

[17] Otsuka F., Byrne R. A., Yahagi K. (2015). Neoatherosclerosis: overview of histopathologic findings and implications for intravascular imaging assessment. *European Heart Journal*.

[18] Nakazawa G., Otsuka F., Nakano M. (2011). The pathology of neoatherosclerosis in human coronary implants bare-metal and drug-eluting stents. *Journal of the American College of Cardiology*.

[19] Loh J. P., Waksman R. (2012). Paclitaxel drug-coated balloons. *JACC: Cardiovascular Interventions*.

[20] Kokkinidis D. G., Prouse A. F., Avner S. J., Lee J. M., Waldo S. W., Armstrong E. J. (2018). Second-generation drug-eluting stents versus drug-coated balloons for the treatment of coronary in-stent restenosis: A systematic review and meta-analysis. *Catheterization and Cardiovascular Interventions*.

[21] Martin D. M., Boyle F. J. (2011). Drug-eluting stents for coronary artery disease: a review. *Medical Engineering & Physics*.

[22] Sehgal S. N. (2003). Sirolimus: its discovery, biological properties, and mechanism of action. *Transplantation Proceedings*.

[23] Huang S., Bjornsti M. A., Houghton P. J. (2003). Rapamycin: mechanisms of action and cellular resistance. *Cancer Biology & Therapy*.

[24] Inoki K., Ouyang H., Li Y., Guan K. L. (2005). Signaling by target of rapamycin proteins in cell growth control. *Microbiology and Molecular Biology Reviews*.

[25] Klümpen H. J., Beijnen J. H., Gurney H., Schellens J. H. M. (2010). Inhibitors of mTOR. *The Oncologist*.

[26] Minacapelli A., Piraino D., Buccheri D., Cortese B. (2017). Drug-coated balloons for the treatment of in-stent restenosis in diabetic patients: A review of currently available scientific data. *Catheterization and Cardiovascular Interventions*.

[27] Sinaga D. A., Ho H. H., Watson T. J. (2016). Drug-coated balloons: a safe and effective alternative to drug-eluting stents in small vessel coronary artery disease. *Journal of Interventional Cardiology*.

[28] Richelsen R. K. B., Overvad T. F., Jensen S. E. (2016). Drug-eluting balloons in the treatment of coronary de novo lesions: A comprehensive review. *Cardiology and Therapy*.

[29] Unverdorben M., Vallbracht C., Cremers B. (2015). Paclitaxel-coated balloon catheter versus paclitaxel-coated stent for the treatment of coronary in-stent restenosis: the three-year results of the PEPCAD II ISR study. *EuroIntervention*.

[30] Scheller B., Hehrlein C., Bocksch W. (2006). Treatment of coronary in-stent restenosis with a paclitaxel-coated balloon catheter. *New England Journal of Medicine*.

[31] Scheller B., Hehrlein C., Bocksch W. (2008). Two year follow-up after treatment of coronary in-stent restenosis with a paclitaxel-coated balloon catheter. *Clinical Research in Cardiology*.

[32] Scheller B., Clever Y. P., Kelsch B. (2012). Long-term follow-up after treatment of coronary in-stent restenosis with a paclitaxel-coated balloon catheter. *JACC: Cardiovascular Interventions*.

[33] Unverdorben M., Vallbracht C., Cremers B. (2009). Paclitaxel-coated balloon catheter versus paclitaxel-coated stent for the treatment of coronary in-stent restenosis. *Circulation*.

[34] Byrne R. A., Neumann F. J., Mehilli J. (2013). Paclitaxel-eluting balloons, paclitaxel-eluting stents, and balloon angioplasty in patients with restenosis after implantation of a drug-eluting stent (ISAR-DESIRE 3): a randomised, open-label trial. *The Lancet*.

[35] Rittger H., Brachmann J., Sinha A. M. (2012). A randomized, multicenter, single-blinded trial comparing paclitaxel-coated balloon angioplasty with plain balloon angioplasty in drug-eluting stent restenosis. *Journal of the American College of Cardiology*.

[36] Xu B., Gao R., Wang J. A. (2014). A prospective, multicenter, randomized trial of paclitaxel-coated balloon versus paclitaxel-eluting stent for the treatment of drug-eluting stent in-stent restenosis. *JACC: Cardiovascular Interventions*.

[37] Toelg R., Merkely B., Erglis A. (2014). Coronary artery treatment with paclitaxel-coated balloon using a BTHC excipient: clinical results of the international real-world DELUX registry. *EuroIntervention*.

[38] Habara S., Mitsudo K., Kadota K. (2011). Effectiveness of paclitaxel-eluting balloon catheter in patients with sirolimus-eluting stent restenosis. *JACC: Cardiovascular Interventions*.

[39] Wöhrle J., Zadura M., Möbius Winkler S. (2012). SeQuent please world wide Registry,prospective registry study. *Journal of the American College of Cardiology*.

[40] Stella P., Belkacemi A., Waksman R. (2011). The valentines trial: results of the first one week worldwide multicentre enrolment trial, evaluating the real world usage of the second generation DIOR paclitaxel drug-eluting balloon for in-stent restenosis treatment. *Euro Intervention*.

[41] Hehrlein C., Dietz U., Kubica J. (2012). Twelve-month results of a paclitaxel releasing balloon in patients presenting with in-stent restenosis first-in-man (PEPPER) trial. *Cardiovascular Revascularization Medicine*.

[42] Alfonso F., Pérez-Vizcayno M. J., Cuesta J. (2018). RIBS IV study investigators (under the auspices of the interventional Cardiology working group of the Spanish society of Cardiology). 3-Year clinical follow-up of the RIBS IV clinical trial: a prospective randomized study of drug-eluting balloons versus everolimus-eluting stents in patients with in-stent restenosis in coronary arteries previously treated with drug-eluting stents. *JACC: Cardiovascular Interventions*.

[43] Windecker S., Kolh P., Alfonso F. (2014). ESC/EACTS guidelines on myocardial revascularization: the task force on myocardial revascularization of the European society of cardiology (ESC) and the European association for cardio-thoracic surgery (EACTS) developed with the special contribution of the European association of percutaneous cardiovascular interventions (EAPCI). *European Heart Journal*.

[44] Cortese B., Di Palma G., Latini R. (2018). Magic Touch®: preliminary clinical evidence with a novel sirolimus drug coated balloon. *Minerva Cardioangiol*.

[45] Cafasso D., Schneider P. (2012). How paclitaxel can improve results in diabetics. *Journal of Cardiovascular Surgery*.

[46] Basoli A., Cametti C., Satriani F. G., Mariani P., Severino P. (2012). Hemocompatibility of stent materials: alterations in electrical parameters of erythrocyte membranes. *Vascular Health and Risk Management*.

[47] Katsanos K., Spiliopoulos S., Kitrou P., Krokidis M., Karnabatidis D. (2018). Risk of death following application of paclitaxel-coated balloons and stents in the femoropopliteal artery of the leg: a systematic review and meta-analysis of randomized controlled trials. *Journal of the American Heart Association*.

[48] Alfonso F., Rivero F., Granada J. F. (2020). Safety of paclitaxel-coated balloons in the coronary arteries. *Journal of the American College of Cardiology*.

[49] Scheller B., Vukadinovic D., Jeger R. (2020). Survival after coronary revascularization with paclitaxel-coated balloons. *Journal of the American College of Cardiology*.

[50] Yerasi C., Case B. C., Forrestal B. J. (2020). Drug-coated balloon for de novo coronary artery disease: JACC state-of-the-art review. *Journal of the American College of Cardiology*.

[51] Hee L., Terluk A., Thomas L. (2017). Late clinical outcomes for SeQuent please paclitaxel-coated balloons in PCI of in-stent restenosis and de novo lesions: a single-center, real world registry. *Catheterization and Cardiovascular Interventions*.

